# Impaired fasting glucose and sulfonylureas increased the risk of major cardiovascular events in patients with inflammatory arthritis

**DOI:** 10.1186/s13098-025-01689-6

**Published:** 2025-04-19

**Authors:** Huan Meng, Ho So, Steven H. Lam, Lai-Shan Tam

**Affiliations:** 1 Department of Medicine and Therapeutics, The Prince of Wales Hospital, The Chinese University of Hong Kong, Shatin, Hong Kong; 2 Liverpool Centre for Cardiovascular Science at University of Liverpool, Liverpool John Moores University, and Liverpool Heart and Chest Hospital, Liverpool, UK

**Keywords:** Rheumatoid arthritis, Psoriatic arthritis, Pre-diabetes, Major adverse cardiovascular events, Anti-diabetic drugs

## Abstract

**Objectives:**

To evaluate the effect of impaired fasting glucose (IFG) and various anti-diabetic agents on the risk of incident major cardiovascular events (MACE) in patients with inflammatory arthritis (IA) including rheumatoid arthritis (RA) and psoriatic arthritis (PsA).

**Methods:**

This was a population-based retrospective cohort study. Patient identification and data retrieval were conducted using a big data platform (The Hospital Authority Data Collaboration Lab) in Hong Kong. Patients with IA were recruited from Jan 2006 to Dec 2015 and followed up until the end of 2018. Time-dependent Cox proportional hazards regression models were used to analyze the association between fasting glucose (FG) levels and anti-diabetic drug use with MACE in IA patients.

**Results:**

A total of 13,905 patients (12,233 RA and 1,672 PsA) were included. 934 patients (6.7%) developed the first MACE after a total of 119,571 patient-years of follow-up. More patients in the MACE group had IFG (FG 5.6–6.9 mmol/l) (19.4% vs. 15.2%, *p* < 0.001) and FG ≥ 7 mmol/l (17.6% vs. 8.1%, *p* < 0.001) at baseline. In the subgroup of patients who were not taking any anti-diabetic medications, a prediabetic state was found to be independently associated with a higher risk of MACE (HR 2.43, 95%CI 1.97–2.99 in CRP model and HR 2.54, 95%CI 1.50–7.71 in ESR model). On the other hand, in patients with diabetes, sulfonylurea use increased the risk of MACE development by 55% (HR 1.55, 95%CI 1.14–2.09) after adjusting for other covariates.

**Conclusions:**

In a large cohort of patients with IA, IFG and sulfonylureas use were found to be independently associated with an increased risk of incident MACE.

**Supplementary Information:**

The online version contains supplementary material available at 10.1186/s13098-025-01689-6.

## Key messages

### What is already known about this subject?

- Patients with inflammatory arthritis (IA) including rheumatoid arthritis (RA) and psoriatic arthritis (PsA) have an increased risk of major cardiovascular events (MACE).
- In the general population, impaired fasting glucose (IFG) was found to be associated with an elevated risk of all-cause mortality as well as incident cardiovascular disease.

### What does this study add?

- IA patients with IFG were found to have a significantly increased risk of incident MACE after adjusting for the effects of inflammatory burden, traditional cardiovascular risk factors and anti-rheumatic drugs.
- The use of sulfonylurea was independently associated with a higher incidence of MACE in IA patients.

### How might this impact on clinical practice or future developments?

- These findings suggest that tight control of blood glucose levels and careful selection of anti-diabetic medication may be important in reducing the risk of MACE in patients with RA and PsA.

## Introduction

Inflammatory arthritis (IA), which includes rheumatoid arthritis (RA) and psoriatic arthritis (PsA), is associated with an increased risk of major adverse cardiovascular events (MACE) [1, 2], with both conditions reportedly having a similar incidence of MACE [3]. They predispose patients to cardiovascular (CV) risk through several mechanisms, including chronic inflammatory burden, the impact of pharmacotherapies, and a high prevalence of traditional CV risk factors such as type 2 diabetes mellitus (T2DM) and hypertension [4, 5]. Approximately half of the elevated cardiovascular disease (CVD) risk in RA can be attributed to traditional CV risk factors [6]. Prediabetes is strongly linked to the development of T2DM, as evidenced by a study of two million adults showing that young individuals diagnosed with prediabetes at age 20 face a 90% lifetime risk of developing diabetes [7]. In the prediabetic state, increased lipolysis, increased β cell dysfunction and decreased endogenous levels of glucagon-like peptide 1 (GLP-1) coincide with aberrant expression of proinflammatory cytokines, creating a detrimental environment for CVD [8–10]. A recent meta-analysis comprising 129 studies revealed that the general population with prediabetes compared to those with normoglycaemia, exhibited an absolute risk difference of 7.36 (95% confidence interval [CI] 9.59–12.51) for all-cause mortality, 8.75 (95%CI 6.41–10.49) for composite CVD per 10,000 person-years [11]. However, the interplay of metabolic and inflammatory effects adds complexity to the interpretation of the association between prediabetes and CVD in IA. Data exploring the effect of prediabetes on the risk of CVD in patients with IA were scanty.

On top of the glucose-lowering effects, different anti-diabetic medications have differential metabolic effects [12, 13]. While metformin, as well as some newer agents, have been shown to be additionally CVD protective in DM patients [14–16], the roles of other commonly used drugs such as sulfonylureas and insulin are more controversial [17–20]. The CV risk associated with sulfonylureas in patients with T2DM can be analyzed from two contrasting perspectives. Sulfonylureas can cause the inhibition of ischaemic conditioning, hypoglycaemia, and weight gain, which have been postulated as potential contributors to an increased susceptibility to major adverse cardiovascular events (MACE) [18]. However, sulfonylureas also exert a glucose-lowering effect [21], thereby mitigating the risk of incident MACE. Studies examining the CVD effect of various anti-diabetic agents in patients with IA taking into account the multitude of contributing factors are lacking.

Thus, the purpose of this study was to evaluate the effect of prediabetes and various anti-diabetic medications overtime on the risk of incident MACE in patients with IA, factoring in multiple clinical variables, including other traditional CV risk factors, markers of systemic inflammation and different anti-rheumatic drugs, using a large real-world database.

## Methods

### Data source and patients

Data were retrieved retrospectively from the Hospital Authority Data Collaboration Lab (HADCL) in Hong Kong, which adopted an electronic health record (EHR) system that captures diagnostic and procedure codes, laboratory tests, and prescription records longitudinally [22, 23]. Patients diagnosed with RA and PsA were recruited from Jan 2006 to Dec 2015 and followed until the end of 2018. The physician’s diagnoses were recorded in the system according to the International Classification of Diseases, 9th Revision, Clinical Modification (ICD9-CM). Inclusion criteria were (1) patients who carried the diagnosis code of RA (ICD9-CM: 714) or PsA (ICD9-CM: 696); (2) without a history of MACE before the diagnosis date of RA or PsA; (3) follow-up periods lasted at least 2 years and RA/PsA diagnostic codes lasted ≥ 6 months; and (4) age of or above 18 at disease onset. The index date was defined as the date when patients first received an RA or PsA diagnosis.

### Definition of impaired fasting glucose (IFG) and diabetes

The prediabetic state was represented by IFG, which was defined as a fasting plasma glucose level between 5.6–6.0 mmol/L, according to the American Diabetes Association [24], in any one available measurement record. People were identified as having a diabetes diagnosis based on the ICD9-CM code or being prescribed at least one antidiabetic medication.

### Outcomes

The outcome was the first occurrence of MACE. MACE was defined as a composite of myocardial infarction (MI), unstable angina, ischemic stroke, hemorrhagic stroke, transient ischemic attack, and CV-related death (diagnosis codes listed in Supplementary Table 1).

### Covariates and medication exposures

Demographic data and traditional CV risk factors (including age, gender, T2DM, hypertension, and dyslipidemia) were retrieved. The definition of CV risk factors at baseline or ever was based on the ICD9-CM and/or by the dispensed prescription records of treatment for these conditions at baseline or during follow-up (details of the treatment items are listed in Supplementary Table 2). FG, glycated hemoglobin A1c (HbA1c), and lipid profile were recorded. The atherogenic index of plasma (AIP) which is the ratio of triglycerides to high-density lipoprotein cholesterol logarithmically transformed was calculated [25]. Inflammatory burden, including arthritis disease duration, and levels of erythrocyte sedimentation rate (ESR) and C-reactive protein (CRP) were also collected. All the covariates except age, gender, and arthritis disease duration were derived yearly after the index date until the end of follow-up. To handle the missing data, we used a complete-case analysis that only used the available data.

The anti-diabetic medication exposures were reported as the use of oral antihyperglycemic agents (OHAs), including metformin, sulfonylureas**,** thiazolidinedione, dipeptidyl peptidase-4 inhibitors (DPP4i), and sodium-glucose linked transporter-2 inhibitors (SGLT2), GLP1 receptor agonists, and the use of insulin. The anti-rheumatic drugs, including conventional synthetic disease-modifying antirheumatic drugs (csDMARDs), biologic DMARDs, non-steroidal anti-inflammatory drugs (NSAIDs), and glucocorticoids were also recorded yearly. All the pharmacotherapies were tracked according to the British National Formulary codes (Supplementary Table 2). Drug prescriptions for combination use were analyzed separately.

### Statistical analysis

Descriptive statistics were used for baseline demographic and clinical characteristics, including frequencies or means ± SD. The baseline covariates were analyzed by the Student’s t-test for continuous variables and the χ^2^ test for categorical variables. Time-dependent cox proportional hazards regression models were conducted to analyze the independent association between the covariates and MACE. All laboratory results were recorded as time-varying covariates with an update in a yearly interval. Each yearly record of FG, lipid profile, ESR and CRP level was calculated by taking the mean values of the available measurements during each particular year. In addition, medications dispensed during follow-up were also assessed, and the association between drugs used and MACE was analyzed at a yearly interval. The users were compared with the non-users. Intervals were discarded from the analysis if there were missing data. Univariable analysis was calculated for covariates and medication variables, and variables with a *p*-value less than 0.05 were included in the multivariate analysis. Further analyses were performed to assess the interactive effect of drug use and FG levels. The Kaplan–Meier curves were used to visualize the CV event-free survival between different groups. Statistical significance was determined using a *p* < 0.05. All analyses were performed in R version 4.0.

## Results

### Clinical characteristics

A total of 13,905 patients, of whom 12,233 with RA and 1672 with PsA, were recruited. The baseline demographic characteristics of the whole cohort are shown in Table 1. After 119,571 patient-years of follow-up, 934 patients (6.7%) developed incident MACE. Patients in the MACE group were older (68.9 ± 11.8 vs. 56.0 ± 14.2 years, *p* < 0.001) and were more likely to be of male gender (27.5% vs. 22.9%, *p* = 0.001). At baseline, a significantly higher proportion of them had FG level 5.6–6.9 mmol/L (19.4% vs. 15.2%, *p* < 0.001), FG level ≥ 7 mmol/l (17.6% vs. 8.1%, *p* < 0.001), or OHAs (15.0% vs 6.8%, *p* < 0.001) and insulin (4.2% vs 1.1%, *p* < 0.001). They also had higher levels of inflammatory markers but were less likely to be on anti-rheumatic medications except for glucocorticoids (Supplementary Table 3). Most IA patients have had their glucose levels checked (95.3%), and the details of patients with no glucose checked and ever glucose checked are shown in Supplementary Table 4.

**Table 1:** Baseline demographic and clinical characteristics, cardiovascular risk factors, and treatments between patients with different inflammatory arthritis

Variables	Entire cohort (n = 13,905)	PsA cohort (n = 1672)	RA cohort (n = 12,233)	p-value
Age, years	56.9 ± 14.4	51.0 ± 12.8	57.7 ± 14.4	< 0.001*
Male, n (%)	3222 (23.2)	942 (56.3)	2280 (18.6)	< 0.001*
Disease duration, years	0.6 ± 1.5	0.3 ± 1.0	0.7 ± 1.5	< 0.001*
Baseline traditional factors				
Diabetes, n (%)	1153 (8.3)	208 (12.4)	945 (7.7)	< 0.001*
Hypertension, n (%)	4834 (34.8)	613 (36.7)	4221 (34.5)	0.087
Dyslipidemia, n (%)	1154 (8.3)	191 (11.4)	963 (7.9)	< 0.001*
CRP, mg/dl	1.68 ± 2.45	1.58 ± 2.40	1.70 ± 2.45	0.068
ESR, mmL/h	45.1 ± 29.9	36.1 ± 27.6	46.4 ± 30.0	< 0.001*
TC/HDL	3.7 ± 1.2	4.2 ± 1.2	3.7 ± 1.2	< 0.001*
LDL/HDL	2.3 ± 1.0	2.6 ± 1.0	2.2 ± 1.0	< 0.001*
Atherogenic index, log (TG/HDL)	− 0.1 ± 0.29	0.0 ± 0.3	− 0.1 ± 0.3	0.014*
FG, mmol/l	5.8 ± 1.7	5.8 ± 1.7	5.9 ± 1.7	0.003*
FG < 5.6 mmol/l, n (%)	4961 (59.5)	605 (57.3)	4356 (60.0)	0.119
FG 5.6–6.9 mmol/l, n (%)	2158 (25.9)	274 (25.9)	1884 (25.9)	0.999
FG ≥ 7 mmol/l, n (%)	1213 (14.6)	177 (16.8)	1036 (14.2)	0.034*
Baseline treatment				
bDMARDs				
Anti-TNF, n (%)	376 (2.7)	95 (5.7)	281 (2.3)	< 0.001*
Non-anti-TNF, n (%)	65 (0.5)	5 (0.3)	60 (0.5)	0.376
csDMARDs				
MTX, n (%)	7714 (55.5)	918 (54.9)	6769 (55.6)	0.634
SLZ, n (%)	2431 (17.5)	285 (17.0)	2146 (17.5)	0.640
LEF, n (%)	1596 (11.5)	141 (0.8)	1455 (11.9)	< 0.001*
NSAIDs				
COXII inhibitors	936 (6.7)	85 (5.1)	851 (7.0)	0.005*
Non-COXII inhibitors	9747 (70.1)	1224 (73.2)	8524 (69.7)	0.004*
Glucocorticoids, n (%)	4782 (34.4)	176 (10.5)	4606 (37.7)	< 0.001*
Statin, n (%)	1090 (7.8)	178 (10.6)	912 (7.5)	< 0.001*
Anti-coagulant, n (%)	34 (0.2)	1 (0.0)	33 (0.3)	0.178
Anti-platelet, n (%)	718 (5.2)	86 (5.1)	632 (5.2)	1.000
OHA, n (%)	1024 (7.4)	188 (11.2)	836 (6.8)	< 0.001*
Insulin, n (%)	186 (1.3)	26 (1.6)	160 (1.3)	0.477

*PsA* psoriatic arthritis, *RA* rheumatoid arthritis, *CRP* c-reactive protein, *ESR* erythrocyte sedimentation rate, *HDL* high-density lipoprotein, *LDL* low-density lipoprotein, *FG* fasting glucose, *bDMARDs* biological disease-modifying anti-rheumatic drugs, *TNF* tumor necrosis factor, *csDMARDs* conventional synthetic disease-modifying anti-rheumatic drugs, *MTX* methotrexate, *SLZ* sulfasalazine, *LEF* leflunomide, *NSAIDs* non-steroidal anti-inflammatory drugs, *COXII* cyclooxygenase-2, *OHA* Oral Hypoglycemic Agents

### IFG and the risk of MACE

**Fig. 1:**
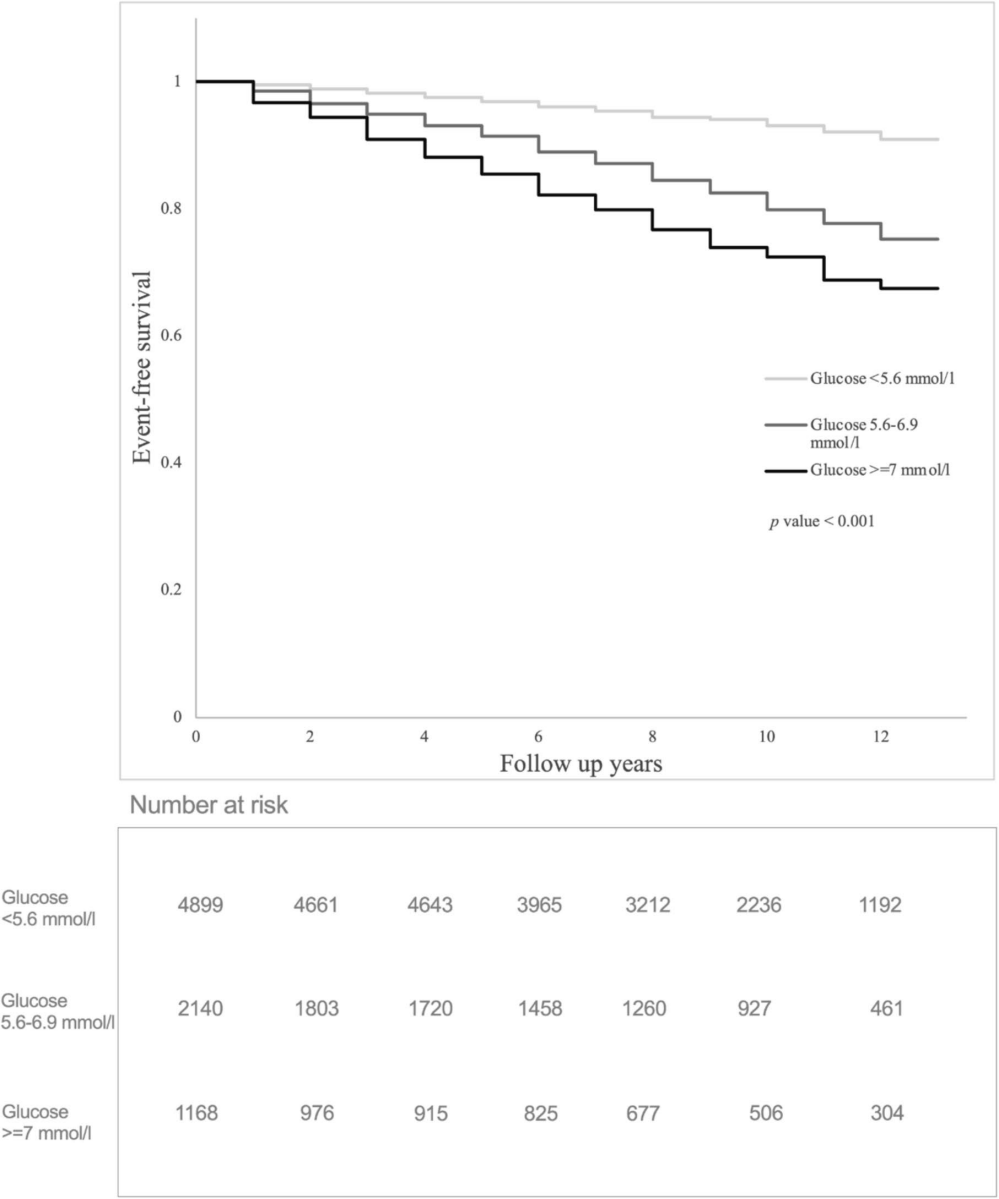
Kaplan–Meier curves and the log-rank test showing the cardiovascular event-free survival among patients with fasting glucose < 5.6 mmol/l or 5.6–6.9 mmol/l or ≥ 7 mmol/l in the entire cohort. Number at risk indicates the number of patient-intervals (person-time) at each time point, not unique patients

In the time-varying ESR model, IFG (hazard ratio [HR] 2.54, 95%CI 1.50–7.71, *p* < 0.001) and FG ≥ 7 mmol/L (HR 4.47, 95%CI 3.25–6.16,* p* < 0.001) were associated with significantly increased the risk of MACE in IA patients without anti-diabetic drugs after controlling for other traditional CV risk factors, systemic inflammation and use of anti-rheumatic drugs. In the time-varying CRP model, IFG (HR 2.43, 95%CI 1.97–2.99, *p* < 0.001) and FG ≥ 7 mmol/l (HR 3.51, 95%CI 2.53–4.86,* p* < 0.001) were also independently associated with an elevated risk of MACE (Table 2). In IA patients who were on anti-diabetic drugs, only FG ≥ 7 mmol/l was independently associated with MACE in the ESR model (HR 1.79, 95%CI 1.18–2.70,* p* < 0.001) and CRP model (HR 1.57, 95%CI 1.05–3.19,* p* = 0.025) (Supplementary Table 5). The event-free survival curves of IA patients with different FG levels are presented in Fig. 1. A significant difference in event-free survival with different FG levels was also noted in a separate analysis of the RA or PsA cohort (both are *p* < 0.001) (Supplementary Fig. 1a and b). Supplementary Figs. 2a and 2b showed the significant differences in different FG levels between IA patients with and without anti-diabetic drugs (*p* < 0.001).

**Table 2:** Multivariable Cox proportional hazards regressions using the demographic variables as time fixed and the other features as time-dependent predictors (being updated at each visit) in patients without any diabetic drug use (RA and PsA cohort)

Variables	Model 1^a^		Model 2^b^	
	Time-dependent HR (95% CI)	*p* value	Time-dependent HR (95% CI)	*p* value
Age	1.06 (1.05–1.07)	< 0.001*	1.06 (1.05–1.07)	< 0.001*
Male	1.57 (1.26–1.95)	< 0.001*	1.31 (1.05–1.62)	0.014*
Disease duration	1.07 (1.01–1.13)	0.013	1.07 (1.01–1.12)	0.017*
Ever hypertension	3.50 (2.58–4.74)	< 0.001*	3.60 (2.65–4.88)	< 0.001*
Time-varying laboratory results				
ESR	1.01 (1.00–1.01)	< 0.001*		
CRP			1.10 (1.08–1.13)	< 0.001*
TC/HDL	0.72 (0.39–1.31)	0.281	0.62 (0.33–1.15)	0.131
LDL/HDL	1.51 (0.79–2.88)	0.211	1.78 (0.92–3.44)	0.089
Atherogenic index log (TG/HDL)	3.40 (1.50–7.71)	0.003*	3.45 (1.52–7.79)	0.003*
FG < 5.6	Ref	NA	Ref	NA
FG 5.6–6.9	2.54 (1.50–7.71)	< 0.001*	2.43 (1.97–2.99)	< 0.001*
FG ≥ 7	4.47 (3.25–6.16)	< 0.001*	3.51 (2.53–4.86)	< 0.001*
Time-varying treatment				
bDMARDs				
Anti-TNF	0.70 (0.40–1.23)	0.214	0.70 (0.40–1.23)	0.214
Non-anti-TNF	0.58 (0.28–1.17)	0.127	0.54 (0.27–1.11)	0.092
csDMARDs				
MTX	0.76 (0.62–0.93)	0.008*	0.78 (0.64–0.96)	0.016*
SLZ	1.18 (0.96–1.45)	0.111	1.15 (0.94–1.41)	0.168
NSAIDs				
COXII inhibitors	0.56 (0.35–0.90)	0.016*	0.65 (0.42–1.01)	0.055
Non-COXII inhibitors	0.83 (0.67–1.01)	0.067	0.81 (0.42–1.00)	0.048*
Glucocorticoids	2.16 (1.77–2.64)	< 0.001*	1.96 (1.61–2.39)	< 0.001*

*NA* not available, *ESR* erythrocyte sedimentation rate, *CRP* c-reactive protein, *bDMARDs* biological disease-modifying anti-rheumatic drugs, *TC* total cholesterol, *HDL* high-density lipoprotein cholesterol, *LDL* low-density lipoprotein cholesterol, *TG* triglycerides, *FG* fasting glucose, *TNF* tumor necrosis factor, *csDMARDs* conventional synthetic disease-modifying anti-rheumatic drugs, *MTX* methotrexate, *SLZ* sulfasalazine, *NSAIDs* non-steroidal anti-inflammatory drugs, *COXII* cyclooxygenase-2

^*^Statistically significant at *p* ≤ 0.05

^a^Adjusted for age, sex, disease duration, ever hypertension, ESR, TC/HDL. LDL/HDL, Atherogenic index log (TG/HDL), bDMARDs, csDMARDs, NSAIDs and glucocorticoids

^b^Adjusted for age, sex, disease duration, ever hypertension, CRP, TC/HDL. LDL/HDL, Atherogenic index log (TG/HDL), bDMARDs, csDMARDs, NSAIDs and glucocorticoids

### Anti-diabetic medications and the risk of MACE

In the univariate analysis, inflammation, traditional CV factors, SLZ, and steroid use were significantly associated with a higher risk of MACE (Supplementary Table 6). The use of anti-diabetic drugs at baseline and during the follow-up is shown in Supplementary Fig. 3. In the cohort of IA patients with diabetes, time-varying sulfonylurea (HR 1.77, 95% CI 1.40–2.25, *p* < 0.001) and insulin use (HR 5.95, 95% CI 4.68–7.57, *p* < 0.001) were associated with increased risk of MACE, whereas metformin was related to reduced risk (HR 0.69 95% CI 0.54–0.88, *p* = 0.003) in the univariate analysis (Supplementary Table 7). After adjustment for other potential covariates, including age, gender, traditional CV risk factors, inflammatory markers, and the use of anti-rheumatic medications, the use of sulfonylurea (HR 1.50, 95%CI 1.09–2.05, *p* = 0.012 in ESR model; HR 1.55, 95%CI 1.14–2.09, *p* = 0.005 in CRP model) and insulin (HR 3.11, 95%CI 2.23–4.34, *p* < 0.001 in ESR model; HR 3.22, 95%CI 2.33–4.45, *p* < 0.001 in CRP model) were significantly associated with increased risk of MACE (Table 3). In the subgroup analysis, insulin users were also found to be older and carrying more adverse CVD risk factors (Supplementary Table 8). On the other hand, the interaction of insulin use and FG ≥ 7 reduced the risk of MACE (HR 0.43, 95%CI 0.19–0.98, *p* = 0.044 in the ESR model; HR 0.38, 95%CI 0.18–0.84, *p* = 0.017 in the CRP model) (Supplementary Table 9).

**Table 3:** Multivariable Cox proportional hazards with time-varying diabetic treatment (being updated at each visit) in patients with ever diabetic cohort

Variables	Model 1^a^		Model 2^b^	
	Time-dependent HR (95% CI)	*p* value	Time-dependent HR (95% CI)	*p* value
Age	1.04 (1.02–1.06)	< 0.001*	1.04 (1.02–1.06)	< 0.001*
Male	1.41 (1.01–1.97)	0.045*	1.11 (0.81–1.53)	0.524
Disease duration	1.01 (0.93–1.10)	0.879	1.02 (0.94–1.10)	0.708
Ever hypertension	2.44 (1.34–4.46)	0.004*	2.57 (1.44–4.57)	0.001*
Time-varying laboratory results				
ESR	1.01 (1.01–1.02)	< 0.001*		
CRP			1.10 (1.07–1.14)	< 0.001*
TC/HDL	1.09 (0.71–1.66)	0.696	1.02 (0.66–1.57)	0.920
LDL/HDL	1.01 (0.57–1.66)	0.982	1.01 (0.62–1.63)	0.983
Atherogenic index log (TG/HDL)	0.98 (0.38–2.54)	0.973	1.07 (0.43–2.71)	0.879
FG < 5.6	Ref	NA	Ref	NA
FG 5.6–6.9	0.81 (0.49–1.34)	0.411	0.80 (0.50–1.28)	0.342
FG ≥ 7	1.31 (0.86–2.02)	0.211	1.20 (0.79–1.81)	0.395
Time-varying treatment				
bDMARDs				
Anti-TNF	1.09 (0.55–2.17)	0.799	1.25 (0.65–2.40)	0.502
Non-anti-TNF	0.53 (0.13–2.17)	0.376	0.45 (0.11–1.85)	0.271
csDMARDs				
MTX	0.74 (0.54–1.01)	0.057	0.80 (0.59–1.10)	0.167
SLZ	0.86 (0.61–1.22)	0.394	0.96 (0.68–1.33)	0.787
NSAIDs				
COXII inhibitors	0.84 (0.45–1.58)	0.593	0.87 (0.46–1.62)	0.659
Non-COXII inhibitors	0.76 (0.54–1.07)	0.117	0.69 (0.49–0.97)	0.003*
Glucocorticoids	1.79 (1.30–2.46)	< 0.001*	1.68 (1.23–2.29)	0.001*
Metformin	0.86 (0.62–1.19)	0.361	0.73 (0.54–1.00)	0.050
Sulfonylurea	1.50 (1.09–2.05)	0.012*	1.55 (1.14–2.09)	0.005*
Insulin	3.11 (2.23–4.34)	< 0.001*	3.22 (2.33–4.45)	< 0.001*

*NA* not available, *ESR* erythrocyte sedimentation rate, *CRP* c-reactive protein, *TC* total cholesterol, *HDL* high-density lipoprotein cholesterol, *LDL* low-density lipoprotein cholesterol, *TG* triglycerides, *FG* fasting glucose, *bDMARDs* biological disease-modifying anti-rheumatic drugs, *TNF* tumor necrosis factor, *csDMARDs* conventional synthetic disease-modifying anti-rheumatic drugs, *MTX* methotrexate, *SLZ* sulfasalazine, *NSAIDs* non-steroidal anti-inflammatory drugs, *COXII* cyclooxygenase-2

^*^Statistically significant at *p* ≤ 0.05

^a^Adjusted for age, sex, disease duration, ever hypertension, ESR, TC/HDL. LDL/HDL, Atherogenic index log (TG/HDL), bDMARDs, csDMARDs, NSAIDs, glucocorticoids, metformin, sulfonylurea, and insulin

^b^Adjusted for age, sex, disease duration, ever hypertension, CRP, TC/HDL. LDL/HDL, Atherogenic index log (TG/HDL), bDMARDs, csDMARDs, NSAIDs, glucocorticoids, metformin, sulfonylurea, and insulin

## Discussion

The present study is the first to concurrently evaluate the impact of IFG and anti-diabetic drug use on the long-term risk of MACE independent of other traditional CV risk factors, inflammatory burden, and anti-rheumatic drug use in a large population of patients with IA. We found that pre-diabetic state and the use of sulfonylureas were risk factors of incident MACE. The findings extend our understanding of the association of glycemic control and CVD in IA, and could inform patient management in clinical practice.

It is known that IA patients exhibit a higher prevalence of T2DM or IFG compared to the general population [26, 27], potentially attributed to the systemic inflammatory burden. Endothelial dysfunction molecular markers, including intercellular adhesion molecule-1 (ICAM-1) and tumor necrosis factor-α (TNF-α), which are pivotal factors in the development and pathophysiology of atherosclerosis [28], exhibit increased levels in individuals with prediabetes [29]. A retrospective study conducted in Hong Kong, utilizing a territory-wide diabetes surveillance dataset and involving 1,630,942 individuals revealed that prediabetes exhibited a statistically significant association with an elevated age- and sex-adjusted HR of 1.13 (95%CI 1.11–1.14) for CVD [30]. Due to the presence of systemic inflammation, it is reasonable to expect that the combination of pre-diabetes and IA would lead to a significantly elevated CVD risk. A meta-analysis of 10 studies confirms that traditional CV risk factors increase the risk of CV events in RA patients, underscoring the importance of managing these risk factors similarly to non-RA patients [31]. Nonetheless, most of these studies did not analyze the effects of inflammation, either in terms of inflammatory markers or disease activity, during the follow-up period [31]. On the contrary, it has been postulated that chronic hyperglycemia could lead to the formation of irreversible advanced glycation end-products (AGEs) affecting the function of long-lived proteins, including cytokines and immunoglobulins, resulting in a “less active” immune system [32, 33]. The hypothesis was supported by a prospective study showing a lower prevalence of RA in patients with T2DM [34]. The salient finding of our study is that patients without any diabetic drug use during follow-up who had IFG carried a 2.5-fold increased risk of MACE over time compared to those with normoglycemia after adjusting for time-varying inflammatory markers, other conventional CV risk factors, and anti-rheumatic medications. Our results highlight the exaggerated CV risk of prediabetes in the background of systemic inflammatory disease, and early blood glucose control may play a crucial role in the prevention of CVD among patients with IA.

An intriguing finding of our study was that the AIP significantly increased the risk of MACE in IA patients not using any diabetic medications. However, this association was no longer statistically significant in patients who had ever used diabetic drugs. This might be attributed by the fact that patients on anti-diabetic medications often have worse metabolic profile and will more likely be prescribed with medications such as statins or antiplatelet agents. These medications could confound the association of AIP and MACE.

Our study also examined the impact of anti-diabetic drugs on MACE in patients with IA. The CV effect of sulfonylureas is still controversial. They have been reported to be associated with a higher risk of MACE compared with other anti-diabetic drugs by two systematic meta-analyses in T2DM [35, 36]. However, a recent systematic meta-analysis, specifically focused on randomized controlled trials, found no significant difference in terms of all-cause mortality and serious adverse events when comparing the use of metformin-SGLT2 inhibitors versus metformin-sulfonylureas [37]. Sulfonylureas lower blood sugar by binding to ATP-sensitive potassium (KATP) channels on pancreatic β-cells leading to the opening of voltage-gated calcium channels, which then triggers the release of insulin-containing secretory granules from the β-cells into the bloodstream [38]. Nonetheless, not only do sulfonylureas affect pancreatic KATP channels, they also impact myocardial KATP channels, potentially disrupting the cellular pathway responsible for myocardial ischemic protection [39]. Our findings are consistent with many of the previous studies, noting an increased risk of MACE in IA patients on sulfonylureas. The approximately 50% elevated risk also appeared higher than the adjusted HR of 1.3 noted in another cohort study comparing MACE in T2DM patients treated with sulfonylureas versus metformin monotherapy, again hinting the contribution of systemic inflammation [40]. However, we did not perform a separate analysis to determine whether a specific medication within the class of sulfonylureas is more likely to increase the risk of MACE.

Several studies [41, 42], but not all [43], reported that patients using exogenous injected insulin had a higher risk of CVD and all-cause mortality compared to combination therapies of OHAs. Hypoglycemia which is commonly associated with insulin therapy could stimulate inflammatory response and increase oxidative stress, leading to increased CVD [44, 45]. Experimental studies have demonstrated that iatrogenic hyperinsulinemia can elevate blood pressure and result in the retention of sodium, water, and uric acid by the kidneys [46]. The ACCORD study was stopped early because intensive glucose lowering was associated with more hypoglycemia, weight gain, and increased all-cause mortality without any MACE benefit in patients with T2DM [42]. In our study, IA patients who were on insulin had increased risk of MACE in the multivariable model. Interestingly, an opposite relationship was observed when an interaction term of insulin use and FG ≥ 7 mmol/l was incorporated into the regression model. It may suggest that the hazard associated with insulin use is driven by excessive glycemic control, consistent with previous studies. Further studies examining the effects of hypoglycemia on CV outcomes in IA patients are encouraged.

There are some limitations in this study. Firstly, our study only identifies associations, and causal relationships cannot be definitively established due to its retrospective observational nature. Secondly, the lack of data regarding patient adherence to prescribed therapies, medication dosages, alternative medications, and other consumed agents restricts our analysis. In addition, certain traditional CV risk factors, including smoking status, obesity, and family history of CVD, were not incorporated into the analysis due to their unavailability in the database. Thirdly, the generalizability of our findings may be limited by the specific population studied (East Asians), which may not represent all patients with RA and PsA. Fourthly, due to the absence of certain clinical parameters, validated disease activity scores could not be included in the analysis. Inflammatory markers were utilized as a surrogate measure for disease activity in their place. Lastly, the usage of some OHAs was relatively small, such as DPP4i (total person-time intervals: 860 years) and thiazolidinedione (total person-time intervals: 165 years), and almost no IA patients were on GLP1 receptor agonists and SGLT2 inhibitors. With future updates of our database, we will have access to additional data that will facilitate a more comprehensive analysis of the effects of these OHAs on CVD outcomes.

In conclusion, IA patients with IFG had an excess risk of incident MACE before they were diagnosed with DM. The use of sulfonylureas was associated with MACE development in DM patients. To optimize CV outcomes in IA patients, close blood sugar monitoring, earlier treatments and careful selection of drugs considering their differential effects on CV risk are recommended.

## Supplementary Information

Supplementary Material 1.

## Data Availability

No datasets were generated or analysed during the current study.

## Declarations

### Patient and public involvement

Patients and/or the public were not involved in the design, or conduct, or reporting, or dissemination plans of this research.
