# Supplementary material for: Impaired fasting glucose and sulfonylureas increased the risk of major cardiovascular events in patients with inflammatory arthritis
Source: Diabetol Metab Syndr. 2025 Apr 19;17:132. doi: 10.1186/s13098-025-01689-6 (PMC12008961; doi:10.1186/s13098-025-01689-6)

| Supplementary table 1. Patient inclusion criteria by ICD9-CM codes and keywords for diagnoses | |
| --- | --- |
| Parameters | ICD9-CM codes |
| ***Rheumatological diagnoses*** |  |
| RA | 714 |
| PsA | 696 |
| ***Major adverse cardiovascular events (MACE)*** | |
| MI | 410, 410.01, 410.02, 410.1, 410.11, 410.12, 410.2, 410.21, 410.22, 410.3, 410.31, 410.32, 410.4, 410.41, 410.42, 410.5, 410.51, 410.52, 410.6, 410.61, 410.62, 410.7, 410.71, 410.72, 410.8, 410.81, 410.82, 410.9, 410.91, 410.92 |
| UA | 411.1 |
| Composite stroke | 430, 431, 432, 433, 434, 435, 436, 437, 438 |
| TIA | 435.9 |
| Ischemic stroke | 434.91 |
| Hemorrhagic stroke | 431 |
| ***Comorbidities*** | |
| Hypertension | 401.9 |
| Diabetes | 250 |
| Dyslipidemia | 272.4 |
| MI, Myocardial infarction; PsA, Psoriatic arthritis; RA, Rheumatoid arthritis; TIA, Transient ischemic attack; UA, Unstable Angina;. | |

| Supplementary table 2. Drug items of interest under specified BNF chapters | |
| --- | --- |
| BNF Chapter | BNF Chapter Description |
| 10.1.3 | ***b/tsDMARDs*** |
|  | Abatacept, Adalimumab, Baricitinib, Certolizumab, Etanercept, Golimumab, Infliximab, Tofacitinib, Tocilizumab |
| 10.1.3 | ***csDMARDs*** |
|  | Methotrexate, Hydroxychloroquine, Leflunomide |
| 10.1.1 | ***NSAIDs*** |
|  | *Selective COXII inhibitors* Etoricoxib, Celecoxib |
|  | *Non-selective COXII inhibitors* Ibuprofen, Naproxen, Diclofenac |
| 6.3.2, 10.1.2 | ***Glucocorticoids*** |
|  | Prednisolone, Methylprednisolone, Dexamethasone, Dexamethasone, Hydrocortisone |
| 2.2, 2.4, 2.5, 2.6 | ***Anti-hypertension drugs*** |
|  | Diuretics, Beta-blockers, ACE inhibitors, Calcium blockers |
| 6.1.1, 6.1.2 | ***Anti-diabetic drugs*** |
|  | *Oral Hypoglycemic Agents (OHAs):* Metformin, Sulfonylurea, DPP4i, Thiazolidinedione, SGLT2 inhibitors, GLP1 receptor agonists  *Insulin* |
| 2.12 | ***Statins*** |
| Others | Cyclosporin (8.2.2), Rituximab (8.2.3), Sulfasalazine (1.5.1) |
| ACE inhibitors, Angiotensin-converting enzyme inhibitor; BNF codes, British National Formulary codes; b/tsDMARDs, biologic/targeted synthetic disease-modifying anti-rheumatic drugs; COXII, Cyclo-oxygenase-2 inhibitor; csDMARDs, conventional synthetic DMARDs; DPP4i, Dipeptidyl peptidase-4 inhibitors; NSAIDs, Nonsteroidal anti-inflammatory drugs. | |

| Supplementary table 3. Comparison of the baseline demographic and clinical characteristics, cardiovascular risk factors and treatments according to the incident major cardiovascular events status in the entire cohort (n=13905). | | | |
| --- | --- | --- | --- |
| Variables | MACE+ve (n=934) | MACE-ve (n=12971) | *p* value |
| Age, years | 68.9 ± 11.8 | ­56.0±14.2 | <0.001* |
| Male, n (%) | 257 (27.5) | 2965 (22.9) | 0.001 |
| Disease duration, years | 1.2 ± 2.0 | 0.6 ± 1.4 | <0.001* |
| ***Baseline traditional cardiovascular factors*** | | | |
| Diabetes, n (%) | 248 (26.6) | 905 (7.0) | <0.001* |
| Hypertension, n (%) | 613 (65.6) | 4221 (32.5) | <0.001* |
| Dyslipidemia, n (%) | 119 (12.7) | 1035 (8.0) | <0.001* |
| CRP, mg/dl | 2.6 ± 3.2 | 1.6 ± 2.4 | <0.001* |
| ESR, mm/hr | 58.5 ± 32.2 | 42.5 ± 29.6 | <0.001* |
| TC/HDL | 4.0 ± 1.4 | 3.7 ± 1.2 | 0.002* |
| LDL/HDL | 2.4 ± 1.1 | 2.2 ± 1.0 | 0.019* |
| Atherogenic index, log (TG/HDL) | -0.0 ± 0.3 | -0.1 ± 0.3 | <0.001* |
| Fasting glucose, mmol/l | 6.5 ± 2.4 | 5.7 ± 1.6 | <0.001* |
| Fasting glucose |  |  |  |
| <5.6 mmol/l, n(%) | 256 (27.4) | 4705 (36.3) | <0.001* |
| 5.6 - 6.9 mmol/l, n(%) | 182 (19.4) | 1976 (15.2) | <0.001* |
| ≥7 mmol/l, n(%) | 164 (17.6) | 1049 (8.1) | <0.001* |
| ***Baseline treatment*** |  |  |  |
| bDMARDs |  |  |  |
| Anti-TNF, n (%) | 9 (1.0) | 367 (2.8) | <0.001* |
| Non-anti-TNF, n(%) | 2 (0.2) | 63 (0.5) | 0.354 |
| csDMARDs |  |  |  |
| MTX, n (%) | 433 (46.4) | 7281 (56.1) | <0.001* |
| SLZ, n (%)  LEF, n (%) | 104 (11.1)  115 (12.3) | 2327 (17.9)  1481 (11.4) | <0.001*  0.438 |
| NSAIDs |  |  |  |
| COXII inhibitors | 34 (3.6) | 902 (7.0) | <0.001* |
| Non-COXII inhibitors | 595 (63.7) | 9152 (70.6) | <0.001* |
| Glucocorticoids, n (%) | 405 (43.4) | 4377 (33.7) | <0.001* |
| Statin, n (%) | 111 (11.9) | 979 (7.5) | <0.001* |
| Anti-coagulant, n (%) | 3 (0.3) | 31 (0.2) | 0.882 |
| Anti-platelet, n (%) | 141 (15.1) | 577 (4.4) | <0.001* |
| OHAs, n (%) | 140 (15.0) | 884 (6.8) | <0.001* |
| Insulin, n (%) | 39 (4.2) | 147 (1.1) | <0.001* |
| *Statistically significant at *p* ≤ 0.05.  bDMARDs, biological disease-modifying anti-rheumatic drugs; CRP, c-reactive protein; COXII, cyclooxygenase-2; csDMARDs, conventional synthetic disease-modifying anti-rheumatic drugs; ESR, erythrocyte sedimentation rate; HDL, high-density lipoprotein; LDL, low-density lipoprotein; LEF, leflunomide; MACE+ve, patients who developed major cardiovascular events during subsequent follow-up; MACE-ve, patients who did not develop major cardiovascular events during subsequent follow-up; MTX, methotrexate; NSAIDs, non-steroidal anti-inflammatory drugs; OHAs, Oral Hypoglycemic Agents; SLZ, sulfasalazine; TNF, tumor necrosis factor. | | | |

| Supplementary table 4. Comparison of the baseline demographic and clinical characteristics between patients with no glucose check and patients with ever glucose check. | | | |
| --- | --- | --- | --- |
| Variables | Never glucose check  (n=647) | Ever glucose check  (n=13258) | *p* value |
| Age, years | 45.9 ± 15.3 | 57.4 ± 14.1 | <0.001* |
| Male, n (%) | 140 (21.6) | 3082 (23.2) | 0.369 |
| Disease duration, years | 0.2 ± 0.9 | 0.6 ± 1.5 | <0.001* |
| ***Baseline traditional CV risk factors*** | | | |
| Diabetes, n (%) | 4 (0.6) | 1149 (8.7) | <0.001* |
| Hypertension, n (%) | 43 (6.6) | 4791 (36.1) | <0.001* |
| Dyslipidemia, n (%) | 2 (0.3) | 1152 (8.7) | <0.001* |
| CRP, mg/dl | 1.0 ± 2.0 | 1.7 ± 2.5 | <0.001* |
| ESR, mm/hr | 32.7 ± 24.0 | 45.7 ± 30.0 | <0.001* |
| TC/HDL | 3.5 ± 1.0 | 3.7 ± 1.2 | 0.470 |
| LDL/HDL | 2.1 ± 0.8 | 2.3 ± 1.0 | 0.442 |
| Atherogenic index, log(TG/HDL) | -0.1 ± 0.3 | -0.1 ± 0.3 | 0.658 |
| ***Baseline treatment*** |  |  |  |
| bDMARDs |  |  |  |
| Anti-TNF, n (%) | 17 (2.6) | 359 (2.7) | 1.000 |
| Non-anti-TNF,n (%) | 2 (0.3) | 63 (0.5) | 0.757 |
| csDMARDs |  |  |  |
| MTX, n (%) | 324 (50.0) | 7390 (55.7) | 0.005* |
| SLZ, n (%) | 121 (18.7) | 2310 (17.4) | 0.434 |
| LEF, n (%) | 43 (6.6) | 1553 (11.7) | <0.001* |
| HCQ, n (%) | 162 (25.0) | 3804 (28.7) | 0.049* |
| NSAIDs |  |  |  |
| COXII inhibitors, n (%) | 48 (7.4) | 888 (6.7) | 0.526 |
| Non-COXII inhibitors, n (%) | 404 (62.4) | 9343 (70.5) | <0.001* |
| Glucocorticoids, n (%) | 165 (25.5) | 4617 (34.8) | <0.001* |
| Anti-coagulant, n (%) | 1 (0.2) | 33 (0.2) | 0.947 |
| Anti-Platelet, n (%) | 6 (0.9) | 712 (5.4) | <0.001* |
| *Statistically significant at *p* ≤ 0.05. bDMARDs, biological disease-modifying anti-rheumatic drugs; COXII, cyclooxygenase-2; CRP, c-reactive protein; csDMARDs, conventional synthetic disease-modifying anti-rheumatic drugs; ESR: erythrocyte sedimentation rate; HDL, high-density lipoprotein; LDL, low-density lipoprotein; LEF, leflunomide; MTX, methotrexate; NSAIDs, non-steroidal anti-inflammatory drugs; SLZ, sulfasalazine. | | | |

| Supplementary table 5. Multivariable Cox proportional hazards regressions using the demographic variables as time fixed and the other features as time-dependent predictors (being updated at each visit) in patients with ever diabetic drug use (RA and PsA cohort) | | | | |
| --- | --- | --- | --- | --- |
|  | Model 1 | | Model 2 | |
| Variables | Time-dependent HR (95% CI) | *p* value | Time-dependent HR (95% CI) | *p* value |
| Age | 1.05 (1.02-1.06) | <0.001* | 1.04 (1.03-1.06) | <0.001* |
| Male | 1.59 (1.14-2.22) | 0.006* | 1.18 (0.86-1.63) | 0.307 |
| Disease duration | 0.99 (0.91-1.08) | 0.821 | 1.01 (0.93-1.09) | 0.841 |
| Ever hypertension | 2.76 (1.52-5.01) | <0.001* | 2.89 (1.63-5.11) | <0.001* |
| ***Time-varying laboratory results*** | | | | |
| ESR | 1.02 (1.01-1.02) | <0.001* |  |  |
| CRP |  |  | 1.13 (1.09-1.16) | <0.001* |
| TC/HDL | 1.12 (0.69-1.82) | 0.646 | 1.02 (0.63-1.66) | 0.940 |
| LDL/HDL | 0.97 (0.57-1.66) | 0.912 | 1.03 (0.60-1.76) | 0.917 |
| Atherogenic index log(TG/HDL) | 1.02 (0.37-2.80) | 0.967 | 1.20 (0.45-3.19) | 0.710 |
| FG <5.6  FG 5.6 - 6.9  FG ≥7 | Ref  0.82 (0.50-1.35)  1.79 (1.18-2.70) | NA  0.441  <0.001* | Ref  0.79 (0.50-1.27)  1.57 (1.05- 3.19) | NA  0.334  0.025* |
| ***Time-varying treatment*** | | | | |
| bDMARDs  Anti-TNF  Non-anti-TNF | 1.00 (0.50-1.98)  0.49 (0.12-2.00) | 0.990  0.318 | 1.16 (0.60-2.23)  0.42 (0.10-1.71) | 0.661  0.224 |
| csDMARDs  MTX  SLZ | 0.67 (0.49-0.92)  0.79 (0.56-1.12) | 0.012*  0.184 | 0.70 (0.52-0.96)  0.88 (0.64-1.23) | 0.025*  0.470 |
| NSAIDs |  |  |  |  |
| COXII inhibitors | 0.89 (0.47-1.66) | 0.703 | 0.89 (0.48-1.66) | 0.714 |
| Non-COXII inhibitors | 0.68 (0.48-0.96) | 0.026* | 0.60 (0.43-0.84) | 0.003* |
| Glucocorticoids | 2.07 (1.50-2.84) | <0.001* | 2.01 (1.48-2.72) | <0.001* |
| *Statistically significant at *p* ≤ 0.05.  †Adjusted for age, sex, disease duration, ever hypertension, ESR, TC/HDL. LDL/HDL, Atherogenic index log (TG/HDL), bDMARDs, csDMARDs, NSAIDs and glucocorticoids.  ‡Adjusted for age, sex, disease duration, ever hypertension, CRP, TC/HDL. LDL/HDL, Atherogenic index log (TG/HDL), bDMARDs, csDMARDs, NSAIDs and glucocorticoids.  bDMARDs, biological disease-modifying anti-rheumatic drugs; COXII, cyclooxygenase-2; CRP, c-reactive protein; csDMARDs, conventional synthetic disease-modifying anti-rheumatic drugs; ESR, erythrocyte sedimentation rate; FG, fasting glucose; HDL, high-density lipoprotein cholesterol; LDL, low-density lipoprotein cholesterol; LEF, leflunomide; MTX, methotrexate; NA, not available; SLZ, sulfasalazine; TC, total cholesterol; TG, triglycerides; TNF, tumor necrosis factor. | | | | |

| Supplementary table 6. Univariate Cox proportional hazards regressions using the demographic variables as time fixed and the other features as time-dependent predictors (being updated at each visit) in the entire cohort. | | | |
| --- | --- | --- | --- |
| Variables | Person-time intervals (years) | Time-dependent HR (95% CI) | *p* value |
| Age | NA | 1.08 (1.08-1.09) | <0.001* |
| Male | NA | 1.41 (1.22-1.63) | <0.001* |
| Disease duration | NA | 1.13 (1.10-1.17) | <0.001* |
| Ever hypertension | NA | 9.53 (7.91-11.49) | <0.001* |
| Ever dyslipidemia | NA | 6.79 (5.95-7.75) | <0.001* |
| ***Time-varying laboratory results*** |  |  |  |
| ESR | 103307 | 1.02 (1.02-1.02) | <0.001* |
| CRP | 103741 | 1.14 (1.14-1.16) | <0.001* |
| ***Time-varying treatment*** |  |  |  |
| bDMARDs  Anti-TNF  Non-anti-TNF | 6056  2570 | 0.50 (0.35-0.74)  0.51 (0.29-0.88) | <0.001*  0.016* |
| csDMARDs  MTX  SLZ  LEF | 64065  29386  18296 | 0.53 (0.46-0.60)  1.26 (1.10-1.45)  0.99 (0.83-1.18) | <0.001*  <0.001*  0.870 |
| NSAIDs |  |  |  |
| COXII inhibitors | 7968 | 0.60 (0.44-0.83) | 0.002* |
| Non-COXII inhibitors | 64178 | 0.51 (0.44-0.58) | <0.001* |
| Glucocorticoids | 31777 | 2.68 (2.36-3.05) | <0.001* |
| *Statistically significant at *p* ≤ 0.05.  bDMARDs, biological disease-modifying anti-rheumatic drugs; COXII, cyclooxygenase-2; CRP, c-reactive protein; csDMARDs, conventional synthetic disease-modifying anti-rheumatic drugs; ESR, erythrocyte sedimentation rate; FG, fasting glucose; HDL, high-density lipoprotein cholesterol; LDL, low-density lipoprotein cholesterol; LEF, leflunomide; MTX, methotrexate; NA, not available; SLZ, sulfasalazine; TC, total cholesterol; TG, triglycerides; TNF, tumor necrosis factor. | | | |

| Supplementary table 7. Univariate Cox proportional hazards regressions with time varying HbA1c and diabetic treatment (being updated at each visit) in the ever diabetic cohort. | | | |
| --- | --- | --- | --- |
| Variables | Person-time intervals (years) | Time-dependent HR (95% CI) | *p* value |
| ***Time-varying laboratory*** |  |  |  |
| HbA1c | 11371 | 0.95 (0.85-1.07) | 0.413 |
| ***Time-varying treatment*** |  |  |  |
| Metformin | 8160 | 0.69 (0.54-0.88) | 0.003* |
| Sulfonylurea | 5799 | 1.77 (1.40-2.25) | <0.001* |
| DPP4i | 860 | 1.35 (0.87-2.09) | 0.182 |
| Thiazolidinedione | 165 | 0.00 (0.00-INF) | 0.988 |
| Insulin | 2067 | 5.95 (4.68-7.57) | <0.001* |
| *Statistically significant at *p* ≤ 0.05. DPP4i, dipeptidyl peptidase 4 inhibitors. | | | |

| Supplementary table 8. Comparison of the baseline demographic and clinical characteristics between patients with no insulin use and patients with ever insulin use in the diabetic cohort. | | | |
| --- | --- | --- | --- |
| Variables | No insulin use  (n=1193) | Ever insulin use  (n=817) | *p* value |
| Age, years | 60.2 ± 11.7 | 64.9 ± 12.5 | <0.001* |
| Male, n (%) | 358 (30.0) | 233 (28.5) | 0.503 |
| Disease duration, years | 0.7 ± 1.6 | 1.0 ± 1.8 | <0.001* |
| ***Traditional CV risk factors*** | | | |
| Diabetes, n (%) | 677 (56.7) | 476 (58.2) | <0.001* |
| Hypertension, n (%) | 859 (72.0) | 665 (81.4) | <0.001* |
| Dyslipidemia, n (%) | 290 (24.3) | 184 (22.5) | 0.382 |
| CRP, mg/dl | 1.8 ± 2.6 | 2.7 ± 3.2 | <0.001* |
| ESR, mm/hr | 46.5 ± 30.1 | 59.0 ± 32.2 | <0.001* |
| TC/HDL | 3.9 ± 1.2 | 4.1 ± 1.5 | 0.089 |
| LDL/HDL | 2.3 ± 0.9 | 2.4 ± 1.1 | 0.152 |
| Atherogenic index, log (TG/HDL) | 0.0 ± 0.3 | 0.0 ± 0.3 | 0.328 |
| FG, mmol/l | 7.0 ± 2.0 | 7.9 ± 3.2 | <0.001* |
| ***Baseline treatment*** |  |  |  |
| bDMARDs |  |  |  |
| Anti-TNF, n (%) | 27 (2.3) | 12 (1.5) | 0.270 |
| Non-anti-TNF,n (%) | 3 (0.3) | 2 (0.2) | 1.000 |
| csDMARDs |  |  |  |
| MTX, n (%) | 654 (54.8) | 403 (49.3) | 0.017* |
| SLZ, n (%) | 203 (17.0) | 112 (13.7) | 0.052 |
| LEF, n (%) | 134 (11.2) | 116 (14.2) | 0.056* |
| HCQ, n (%) | 285 (23.9) | 178 (21.8) | 0.296 |
| NSAIDs |  |  |  |
| COXII inhibitors, n (%) | 72 (6.0) | 35 (4.3) | 0.106 |
| Non-COXII inhibitors, n (%) | 849 (71.2) | 514 (62.9) | <0.001* |
| Glucocorticoids, n (%) | 389 (32.6) | 352 (43.1) | <0.001* |
| Anti-coagulant, n (%) | 4 (0.3) | 4 (0.5) | 0.858 |
| Anti-Platelet, n (%) | 111 (9.3) | 123 (15.0) | <0.001* |
| Metformin, n (%) | 479 (40.2) | 310 (37.9) | 0.343 |
| Sulfonylurea, n (%) | 375 (31.4) | 320 (39.2) | <0.001* |
| DPP4i, n (%) | 20 (1.7) | 16 (2.0) | 0.767 |
| Thiazolidinedione, n (%) | 2 (0.2) | 6 (0.7) | 0.105 |
| SGLT2 inhibitors, n (%) | 0 (0.0) | 0 (0.0) | NA |
| GLP1 receptor agonists, n (%) | 0 (0.0) | 0 (0.0) | NA |
| *Statistically significant at *p* ≤ 0.05.  bDMARDs, biological disease-modifying anti-rheumatic drugs; COXII, cyclooxygenase-2; CRP, c-reactive protein; csDMARDs, conventional synthetic disease-modifying anti-rheumatic drugs; DPP4i, dipeptidyl peptidase 4 inhibitors; ESR, erythrocyte sedimentation rate; FG, fasting glucose; GLP1 receptor agonists, glucagon-like peptide 1 receptor agonists; HDL, high-density lipoprotein cholesterol; LDL, low-density lipoprotein cholesterol; LEF, leflunomide; MTX, methotrexate; NA, not available; SGLT2 inhibitors, sodium-glucose co-transporter 2 inhibitors; SLZ, sulfasalazine; TC, total cholesterol; TG, triglycerides; TNF, tumor necrosis factor. | | | |

| Supplementary table 9. Multivariable Cox proportional hazards with time varying diabetic treatment and interaction between glucose and insulin in patients with ever diabetic cohort. | | | | |
| --- | --- | --- | --- | --- |
|  | Model 1 | | Model 2‡ | |
| Variables | Time-dependent HR (95% CI) | *p* value | Time-dependent HR (95% CI) | *p* value |
| Age | 1.04 (1.02-1.06) | <0.001* | 1.04 (1.02-1.06) | <0.001* |
| Male | 1.39 (1.00-1.95) | 0.053 | 1.09 (0.79-1.50) | 0.591 |
| Disease duration | 1.01 (0.93-1.10) | 0.859 | 1.02 (0.94-1.10) | 0.704 |
| Ever hypertension | 2.41 (1.32-4.41) | 0.004* | 2.54 (1.43-4.53) | 0.002* |
| ***Time-varying laboratory results*** | | | | |
| ESR | 1.01 (1.01-1.02) | <0.001* |  |  |
| CRP |  |  | 1.11 (1.07-1.14) | <0.001* |
| TC/HDL | 1.10 (0.72-1.69) | 0.649 | 1.03 (0.67-1.59) | 0.886 |
| LDL/HDL | 1.00 (0.61-1.62) | 0.987 | 1.00 (0.62-1.63) | 0.987 |
| Atherogenic index log(TG/HDL) | 0.94 (0.36-2.45) | 0.905 | 1.04 (0.41-2.63) | 0.928 |
| FG <5.6  FG 5.6 - 6.9  FG ≥7 | Ref  1.10 (0.59-2.05)  1.82 (1.04-3.21) | NA  0.755  0.037* | Ref  1.13 (0.61-2.09)  1.80 (1.02-3.16) | NA  0.699  0.041* |
| ***Time-varying treatment*** |  |  |  |  |
| bDMARDs  Anti-TNF  Non-anti-TNF | 1.11 (0.56-2.21)  0.55 (0.13-2.24) | 0.759  0.401 | 1.27 (0.66-2.44)  0.47 (0.11-1.91) | 0.473  0.289 |
| csDMARDs  MTX  SLZ | 0.74 (0.54-1.01)  0.86 (0.61-1.22) | 0.058  0.122 | 0.81 (0.59-1.10)  0.96 (0.69-1.34) | 0.174  0.814 |
| NSAIDs |  |  |  |  |
| COXII inhibitors | 0.84 (0.45-1.58) | 0.588 | 0.88 (0.47-1.63) | 0.678 |
| Non-COXII inhibitors | 0.76 (0.54-1.08) | 0.122 | 0.69 (0.49-0.98) | 0.035* |
| Glucocorticoids | 1.78 (1.29-2.45) | <0.001* | 1.67 (1.22-2.28) | 0.001* |
| Metformin | 0.85 (0.62-1.19) | 0.332 | 0.72 (0.53-0.99) | 0.041* |
| Sulfonylurea | 1.47 (1.09-2.05) | 0.016* | 1.53 (1.13-2.07) | 0.006* |
| Insulin | 6.36 (3.05-13.29) | <0.001* | 7.01 (3.49-14.08) | <0.001* |
| Insulin X FG 5.6-6.9 | 0.40 (0.14-1.15) | 0.089 | 0.40 (0.15-1.06) | 0.065 |
| Insulin X FG≥7 | 0.43 (0.19-0.98) | 0.044* | 0.38 (0.18-0.84) | 0.017* |
| *Statistically significant at *p* ≤ 0.05.  †Adjusted for age, sex, disease duration, ever hypertension, ESR, TC/HDL. LDL/HDL, Atherogenic index log (TG/HDL), bDMARDs, csDMARDs, NSAIDs, glucocorticoids, metformin, sulfonylurea, and insulin.  ‡ Adjusted for age, sex, disease duration, ever hypertension, CRP, TC/HDL. LDL/HDL, Atherogenic index log (TG/HDL), bDMARDs, csDMARDs, NSAIDs, glucocorticoids, metformin, sulfonylurea, and insulin. NA, not available; ESR, erythrocyte sedimentation rate; CRP, c-reactive protein; FG, fasting glucose; bDMARDs, biological disease-modifying anti-rheumatic drugs; TNF, tumor necrosis factor; csDMARDs, conventional synthetic disease-modifying anti-rheumatic drugs; MTX, methotrexate; SLZ, sulfasalazine; LEF, leflunomide; COXII, cyclooxygenase-2. | | | | |

Supplementary figure 1. Kaplan–Meier curves and the log-rank test showing the cardiovascular event-free survival among patients with fasting glucose <5.6mmol/l or 5.6-6.9 mmol/l or ≥7 mmol/l in the (a) RA cohort, (b) PsA cohort. Number at risk indicates the number of patient-intervals (person-time) at each time point, not unique patients.


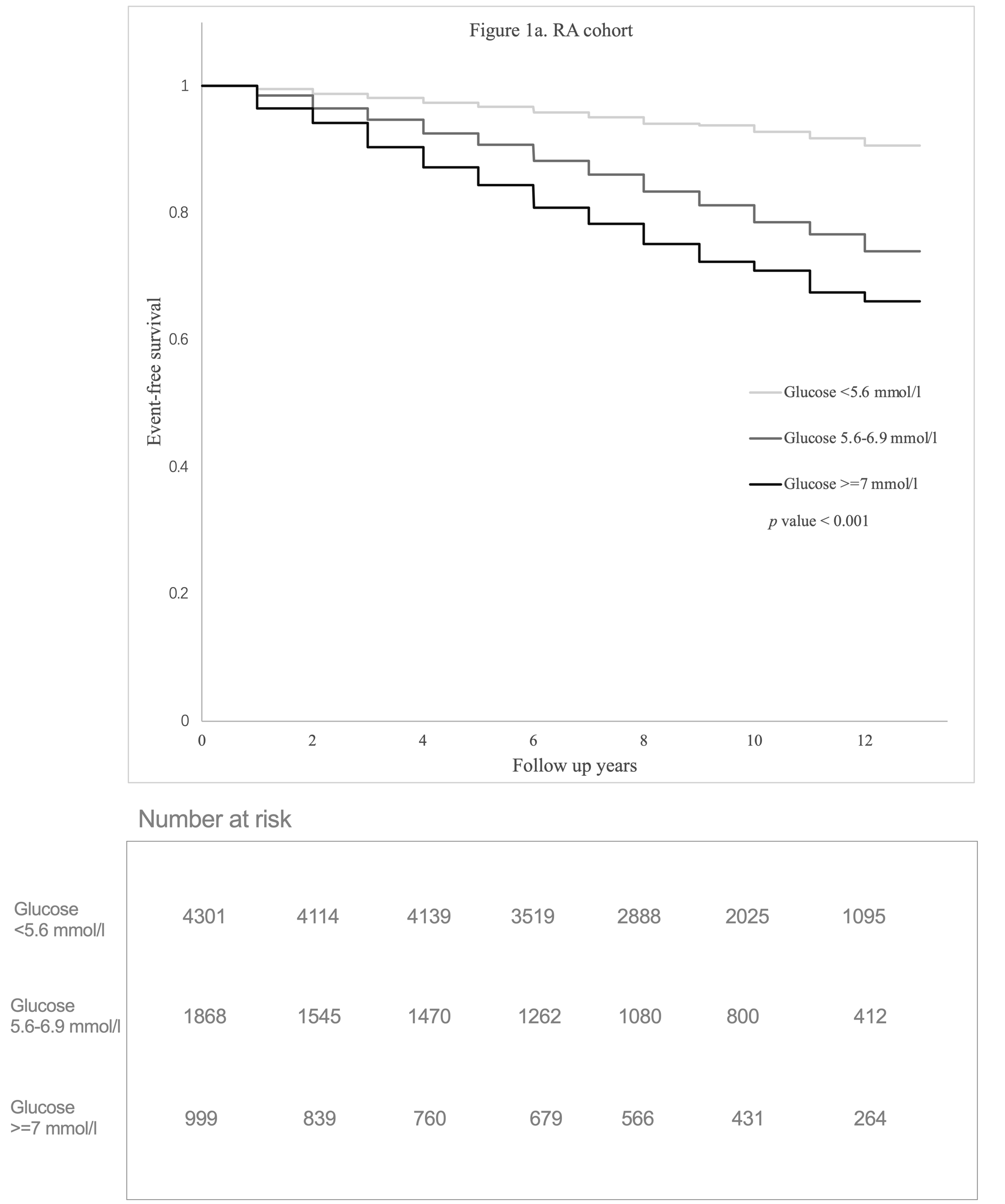


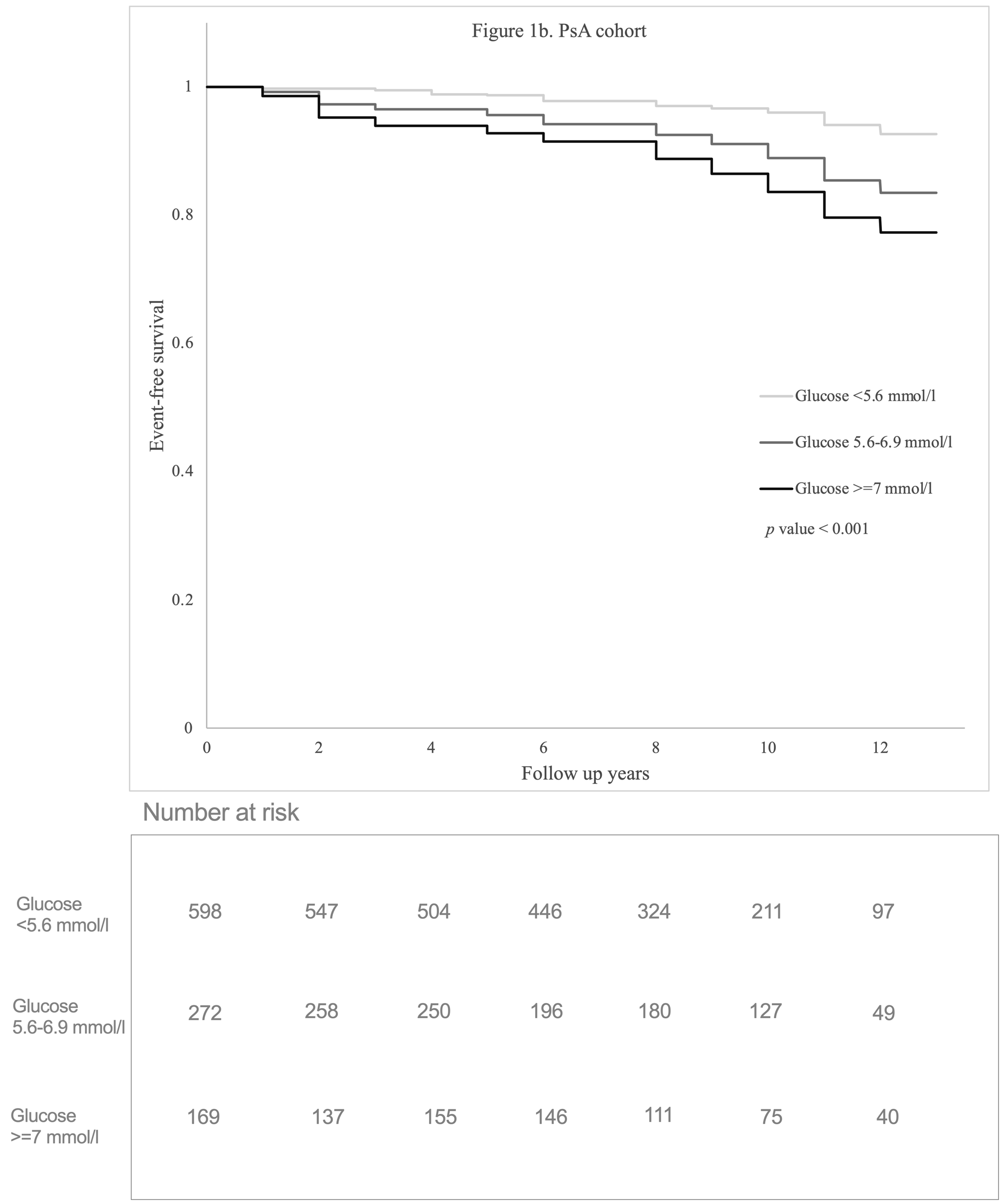


Supplementary figure 2. Kaplan–Meier curves and the log-rank test showing the cardiovascular event-free survival among patients with fasting glucose <5.6mmol/l or 5.6-6.9 mmol/l or ≥7 mmol/l in (a) patients without any use of the diabetic drug, (b) patients with diabetic drug use. Number at risk indicates the number of patient-intervals (person-time) at each time point, not unique patients.


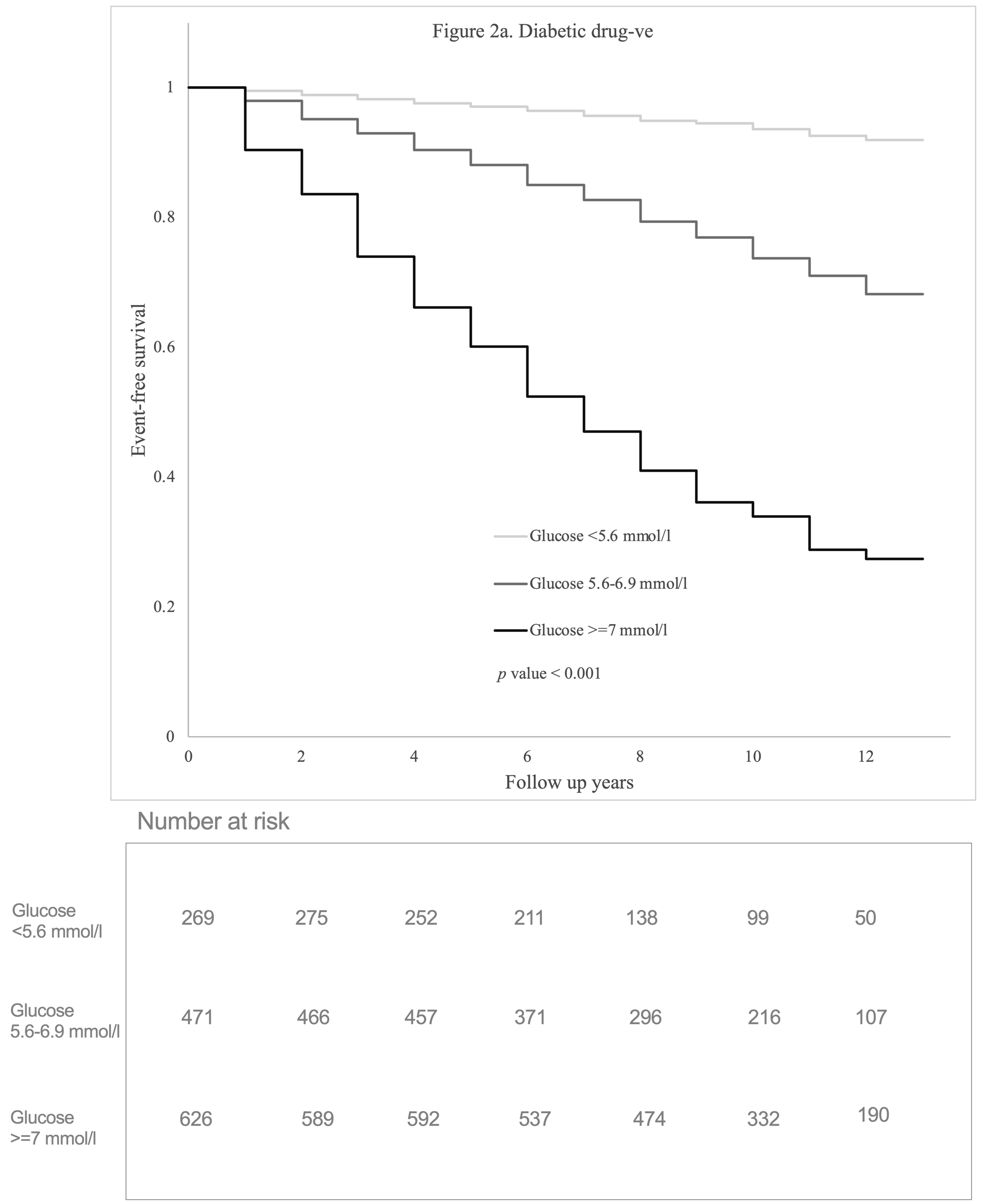


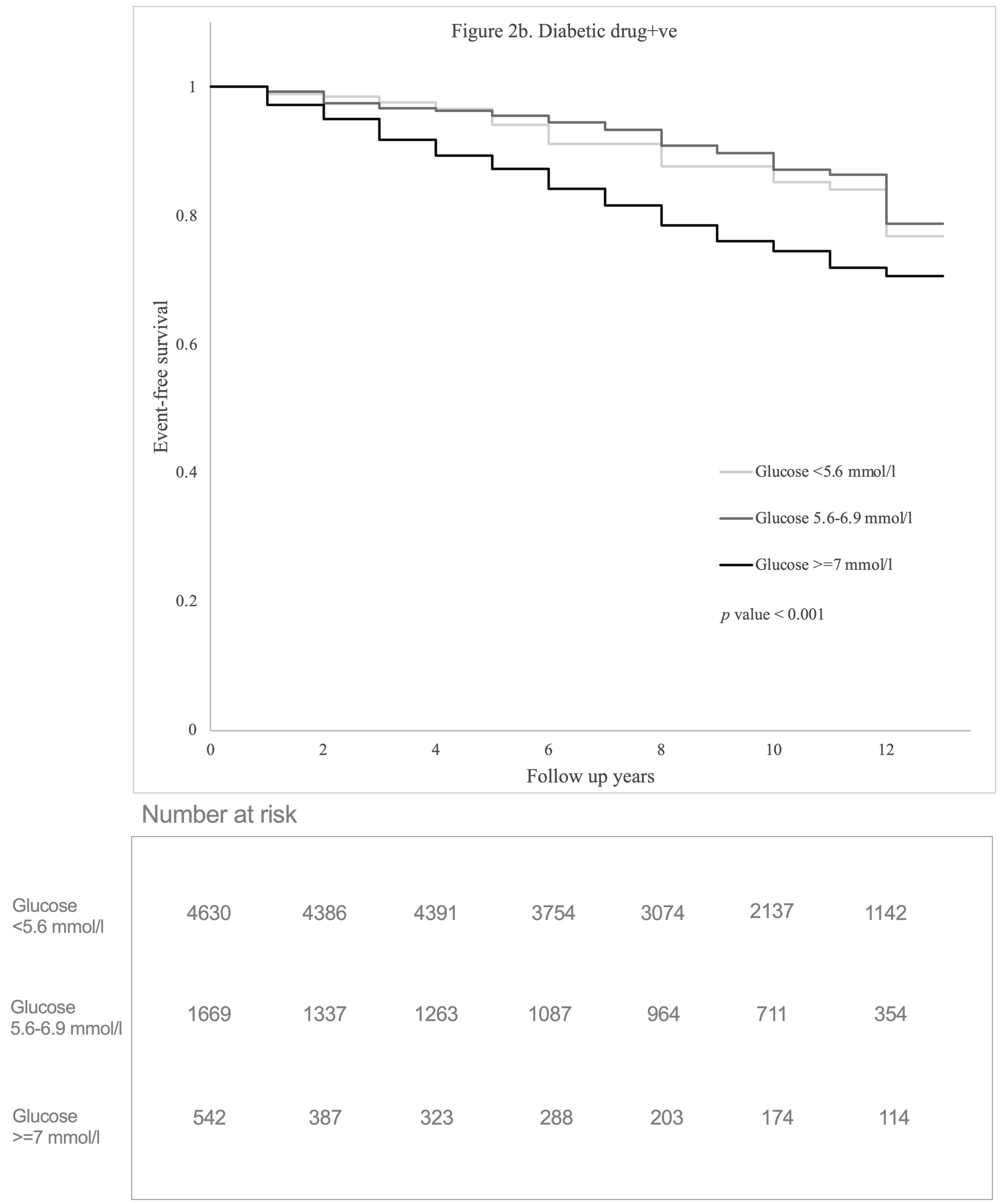


Supplementary figure 3. Baseline and ever use of glucose-lowering drugs in the diabetic cohort.


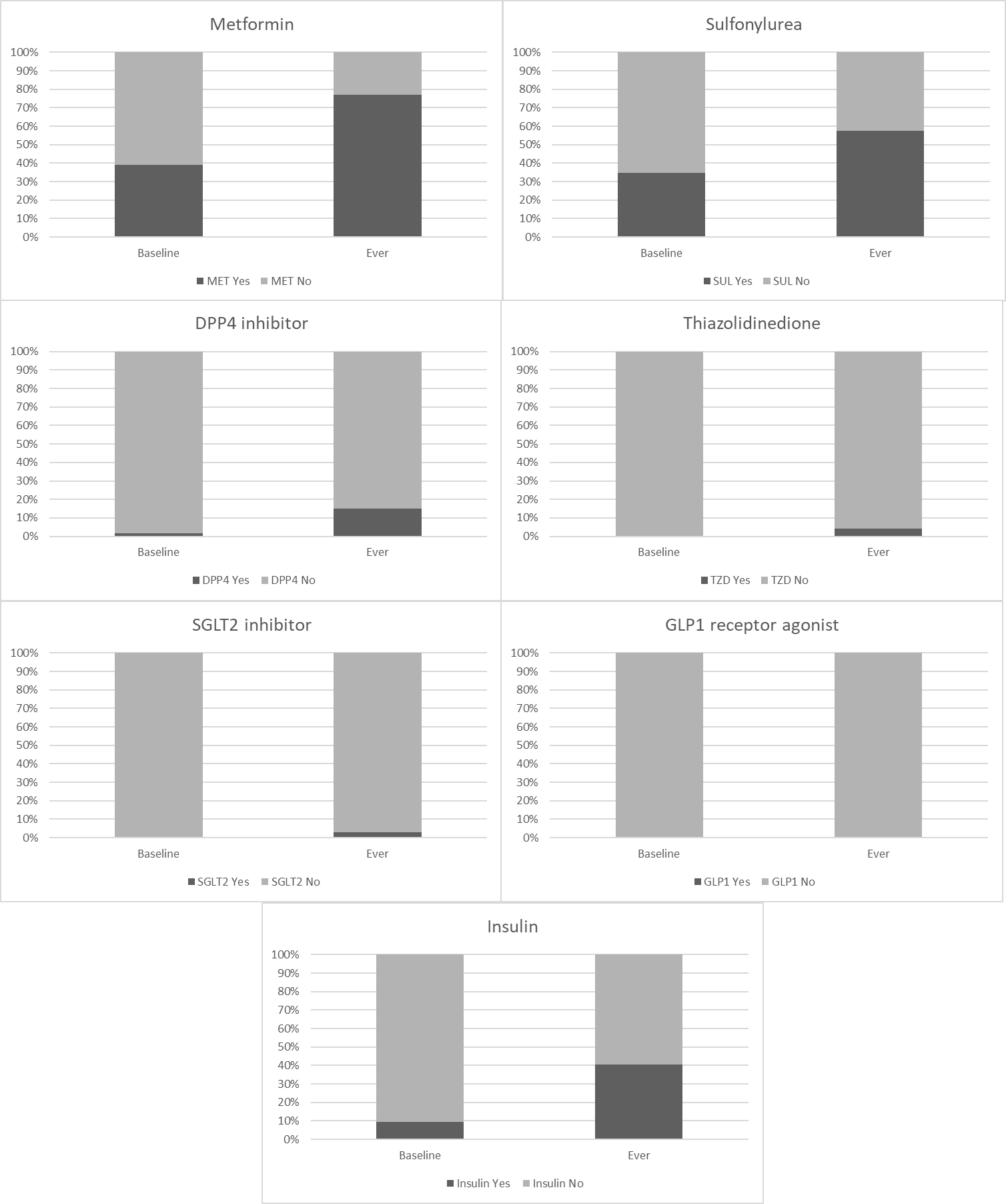

Supplement: Supplementary file 1 — Supplementary Material 1. [file 13098_2025_1689_MOESM1_ESM.docx]
